# Auricularia polytricha aqueous extract supplementation decreases hepatic lipid accumulation and improves antioxidative status in animal model of nonalcoholic fatty liver

**DOI:** 10.7603/s40681-014-0012-3

**Published:** 2014-08-04

**Authors:** Wan-Chun Chiu, Hsu-Hui Yang, Shu-Chi Chiang, Yu-Xuan Chou, Hui-Ting Yang

**Affiliations:** 1 School of Nutrition and Health Sciences, Taipei Medical University, Taipei, Taiwan; 2 Council of Agriculture, Executive Yuan, Fengshan Tropical Horticultural Experimental Branch, Kaohsiung, Taiwan; 3 Department of Nutrition, China Medical University, 91 Hsueh-Shih Road, Taichung 404, Taichung, Taiwan

**Keywords:** Auricularia polytricha, Non-alcoholic fatty liver disease, Two-hit theory

## Abstract

Background: Amelioration effect of Auricularia polytricha water extract (AP) on hepatic injury in an animal model of NAFLD was investigated.

Methods: Forty six-week-old Wistar rats were housed and thirty-two fed ten percent lard high-fat diet to induce NAFLD. After eight weeks of induction, animals were divided into five groups of eight rats each: normal control, high-fat diet, RN (reversion to a normal diet), 1× AP (normal diet plus 0.75% AP, w/w), and 2×AP (normal diet plus 1.5% AP). Animals were sacrificed four weeks later.

Results: Rats receiving either 0.75% or 1.5% AP exhibited effective interruption of NAFLD progression, as evidenced by decreased lipid accumulation and elevated antioxidative status.

Histological examination proved AP anti-inflammatory function and lower level of related markers for tumor necrosis factor-α and interleukin-6. Besides abundant polysaccharides against lipid accumulation, AP had a specific high level of phenolic compounds and tannins thus may be a potent anti-inflammatory and antioxidative agent.

Conclusion: Findings suggest that under normal diet recovery, AP supplement may represent novel, protective material against NAFLD by attenuating inflammatory response, oxidative stress and lipid deposition.

## 1. Introduction

Non-alcoholic fatty liver disease (NAFLD) involves progressive liver damage mainly caused by high dietary intake of cholesterol and saturated fat [1]. Ludwig and colleagues coined the term upon discovering macrovesicular lipid droplets, cell necrosis, inflammation, and sinusoidal fibrosis in 20 female diabetics who were not habitual alcohol consumers [2]. Besides non-alcoholic steatohepatitis (NASH), fatty liver, fibrosis, and cirrhosis are all part of NAFLD. Insulin resistance, oxidative stress, and inflammation play key roles in NAFLD progression [3], yet diverse etiologies exist. Most widely accepted is a two-hit hypothesis [4]: [5] obesity, hyperlipidemia, and diabetes induce hepatic fat accumulation; [6] fat amassed results in lipid peroxidation in hepatic cell membranes, releasing proinflammatory cytokines and activating stellate cells. The double invasion contributes to a series of immune responses: e.g., permeable fat infiltration, inflammatory response, cell necrosis, apoptosis. In brief, two-hit hypothesis entails hepatic fat deposition and lipid peroxidation due mainly to unbalanced nutrient intake. Dietary adjustment or specific functional food supplementation, it is believed, can benefit patients by postponing or even reversing pathological progression [7].

It is well known that edible mushrooms are low in calories and rich in polysaccharides, proteins, vitamins, and minerals. Recently, researchers have paid greater attention to the value of edible mushrooms in food therapy. The family *Auriculae* has two well-known mushrooms, *Auricularia auricula-judae* (AA) and *Auricularia polytricha* (AP). AP is a common edible mushroom in Taiwan. Unlike AA, AP has a thicker fruiting body with very short and fine fur on its backside. Plants of the *Auriculae* genus have abundant functional components: polysaccharides, polyphenols, tannins, etc. [8]. Polysaccharides, especially soluble ones, are primary active components of AP and AA, as discussed in previous studies [9, 10]. In addition to polysaccharides, polyphenols and tannins are also important elements in the genus. Moreover, early research mostly focused on the health-promoting effects of AA, with little discussion of the function of AP. Recently, antitumor effect, immunomodulation, and free radical scavenging of AP was investigated [11-13], unearthing evidence that active components of AP might have capacity to protect against the two-hit theory of NAFLD. Our laboratory analysis indicates AP aqueous extract (AP) having more active compounds and stronger radical-scavenging abilities than AA aqueous extract (Table 1) and water extraction producing less toxic and concentrated or elevated active components of AP. Consequently, we use AP as functional supplement on hepatic injury with animal model of NAFLD.

**Table 1:** Component analysis and antioxidative capacity of *Auricularia Polytricha* and *Auricularia Auricula* water extracts.

	Total protein (mg/g)	Total sugar (mg/g)	Total phenol (mg gallic acid /g)	Tannin (ìg/g)	FRAP (mM Vit C/ g )	TEAC (mM Vit E/g)
AP	105.0± 2.3	593.2± 1.5	10.5± 0.0	764.2± 35.5	17.3 ± 0.4	50.4 ± 2.5
AA	81.7± 0.9	683.9± 1.6	5.4± 0.0	416.4± 9.9	7.5 ± 0.3	32.6 ±1.7

Data based on triplicate analysis from triple sampling of AA and AP extracts, values presented as mean± standard deviation. Abbreviations: AP, Auricularia Polytricha; AA, Auricularia auricular; FRAP, ferric reducing antioxidant power; TEAC, Trolox equivalent antioxidant capacity.

## 2. Materials and methods

### 2.1. Chemicals and Reagents

For biochemical analysis, DiaSys system kits for Hitachi 917 determined serum lipids, hepatic and renal function. (Holzheim, Germany). Commercial kit to gauge fasting blood glucose (FBS) was obtained from Randox Laboratories, Ltd. (Randox Laboratories, London, UK). Blood glucose test strips monitored FBS during experiments. (EasiCheck, Taiwan). Bone-specific alkaline phosphatase (BSAP) was quantified by commercially available, enzyme-linked immunosorbent assay (ELISA) kit (USCNK Life Sciences, Houston, TX). Osteoprotegerin was determined by ELISA kit (Immuno Diagnostic Systems, Boldon, UK).

### 2.2. Plant Extract Preparation

A commercially cultivated strain of AP was purchased from Jhongpu Township, Chiayi, Taiwan, extraction procedure modified from that of Puttaraju and colleagues [13]. Briefly, rehydrated fruiting bodies were steeped in reverse osmosis (RO) water (5 times sample volume) at 126 ºC, high pressure (1.2kg/cm^2^) for 30 min, and ultrasonicated for 1 h. After proper filtration (130-140 mesh), AP was spray-dried and ground to fine powder (0.4mm).

### 2.3. Animals and Treatment

Forty six-week-old, male Sprague-Dawley rats were purchased from the National Laboratory Animal Center (Taipei, Taiwan). After a week of acclimation, 32 animals were fed a high-fat diet containing 88% laboratory rodent chow, 10% lard, and 2% cholesterol, to induce NAFLD [14]. The remaining eight rats were fed a laboratory rodent chow as normal controls. After eight weeks’ induction, thirty-two rats were divided into groups: high-fat diet (HFD), reversion to rodent chow diet (NR), 1× AP (0.75% AP in chow diet, w/w), and 2× AP (1.5% AP in chow diet, w/w). Animals were subsequently fed designated diets for four weeks, then sacrificed; blood and liver samples were collected after study. The Institutional Animal Care and Use Committee of China Medical University approved animal protocols.

### 2.4. Plasma and Liver Analysis

Commercial kit (DiaSYS respons®, Germany) analyzed aspartate aminotransaminase (AST) and alanine ami notransaminase (ALT). AST catalyzed L-aspartate and α-ke toglutarate to form oxaloacetate and L-glutamate, while oxaloacetate and NADH together formed NAD^+^ colored and detected at 340 nm. Pyruvate, end product of ALT catalyzation, formed detectable NAD+ with NADH at 340 nm.

### 2.5. Total cholesterol (plasma, liver), high-density lipoprotein cholesterol (HDL-C), low-density lipoprotein cholesterol (LDL-C), and very LDL cholesterol (VLDL-C)

To obtain HDL-C and LDL-C fractions, we used precipitating reagent to remove VLDL-C, and chylomicron fractions from plasma (HDL precipitant, catalog no. 135409990885, LDL precipitant, catalog no. 143309990885, DiaSYS respons®). For hepatic cholesterol, 2 mL of extraction reagent (chloroform: methanol 2: 1, v/v) was added to 0.5 g of tissue samples and homogenized. Ten-milliliter extracts were vacuumed-dried and concentrated. A commercial kit (CH201, Randox, UK) detected cholesterol concentration of specimens. One milliliter of working solution was added to prepared specimens; absorbance was read at 500 nm and 37 °C, VLDL-C calculated as total cholesterol-(HDL-C + LDL-C).

### 2.6. Plasma and hepatic triglyceride concentrations

Ten-microliter plasma samples were directly treated with 1 mL of working reagent (TR213, Randox), absorbance read at 500 nm after 5-min incubation at 37 °C. Hepatic tissue samples were first extracted with a proper amount of solvent (chloroform: methanol 2: 1, v/v) and Triton x-100 added. Extracts were vacuumed and reconstituted with the working reagent (TR213, Randox). Subsequent procedures were the same as those for serum samples [15].

### 2.7. Blood glucose and insulin levels

During the experiment, glucose meter and blood glucose test strips (TD-4207, EasiCheck, Taiwan) detected fasting blood glucose. A commercial enzyme-linked immunosorbent assay (ELISA) kit for insulin (catalog no. 10-1250-01, Mercodia, Sweden) was used for specimen detection. Briefly, sample (either serum or tissue homogenate) and standards were incubated in a 96-well plate with certain primary antibodies. The secondary antibodies, horseradish peroxidase-avidin, and chromogenic substrate 3,3’,5,5’-tetramethylbenzidine, were added and mixed, data read at OD of 450 nm. HOMA-IR index was calculated as fasting glucose (mmol/L)×fasting insulin (U/mL)/22.5.

### 2.8. Lipid peroxidation

Lipid peroxidation was adapted from Mihara et al. [16]: 100 microliters of serum or liver homogenates placed in a glass tube and mixed with 0.22% sulfuric acid (catalog no. 320501, Sigma-Aldrich, USA), 10% phosphotungstic acid (catalog no. P4006, Sigma-Aldrich), and 0.67% thiobarbituric acid (catalog no. T5500, Sigma-Aldrich). 1-Butanol (catalog no. 360465, Sigma-Aldrich) was added after 95 °C water bath for 1 h, supernatant absorbance read by fluorometer (excitation 515 nm, emission 555 nm), results based on standard curve.

### 2.9. Plasma level of vitamin C

Plasma (100 microliters) was added to 900 μL methanol, then mixed well at 4 °C. Supernatant collected after centrifuge at 3000 rpm was filtered and injected into high-performance liquid chromatography (HPLC, with L-7100 pump and L-7420 UV-VIS detector, Hitachi, Japan) for analysis. Mobile phase contained methanol, deionized water, and glacial acetic acid (80: 17.5: 2.5); C-18, 5-μM, 25-cm column (CA#:581325-U, Ascentis, UK) was used for analysis at 254 nm.

### 2.10. Plasma and liver vitamin E concentrations

Proper 50 μg/mL DL-α-tocopheryl acetate amounts (internal control, catalog no. T3376, Sigma) and 600 mL hexane (catalog no. 650552, Sigma-Aldrich) added to 200 μL of plasma samples or brain homogenates were mixed well. Superscripts were collected after centrifuge at 10,000 rpm for 10 min, methanol (350 μL) added to reconstitute vacuum-dried samples, 80 microliters of filtrate injected into HPLC (with an L-7100 pump and L-7420 UV-VIS detector, Hitachi) for determination at 290 nm. Analytical mobile phase contained methanol (catalog no. 34860, Sigma-Aldrich, US) and deionized water at 98:2 ratio. A C-18, 5 mM, 250-cm × 4.6-mm column (CA#:581325-U, Ascentis, UK ) was used for separation.

### 2.11. Free fatty acids (FFAs)

This study used an FFA assay kit (catalog no. 700310, Cayman, USA). FFAs in plasma formed acyl CoA after catalyzation by acyl CoA synthetase. Acyl CoA oxidase subsequently oxidized acyl CoA to hydrogen peroxide. Product generated fluorescent resorrufin detectable at excitation 530 nm and emission 585 nm.

### 2.12. Superoxide dismutase (SOD)

This study used a SOD assay kit (catalog no. 706002, Cayman). Tetrazolium salt reacts with superoxide and needs SOD to form superoxide; it forms yellow formazan dye at 450 nm. Absorbance represents clearance of superoxide by SOD, expressed as U/mg protein.

### 2.13. Catalase

Our study used a catalase assay kit (catalog no. 707002, Cayman). As catalase catalyzes hydrogen peroxide to water, remaining hydrogen peroxide reacts with methanol and Purpald (4-amino-3-hydrazino-5-mercapto-1, 2, 4-trizole) to yield purple-colored bicyclic aldehyde. The complex was detected at 540 nm, expressed as μmol/min/mg protein to reflect catalase activity in liver tissues.

### 2.14. Glutathione reductase (GR)

A GR assay kit (catalog no. 703202, Cayman) was used for analysis. As GR catalyzes oxidized GSSG to reduced form, GSH, colored NADPH forms colorless NADP^+^ at 340 nm. Decrease in absorbance represents GR activity, value expressed as nmol/min/mg protein.

### 2.15. Glutathione peroxidase (GPx)

A GPx assay kit (catalog no. 703102, Cayman) served for analysis. Basically, reduced glutathione (GSH) in the liver turned into oxidized form whenever GPx catalyzed hydrogen peroxide to water. Decreasing rate of NADPH was measured at 340 nm, whenever oxidized GSSG returned to its reduced form, value expressed as nmol/min/mg protein.

### 2.16. Plasma level of interleukin (IL)-6

A rat IL-6 Platinum ELISA kit (catalog no. BMS625TWO, BenderMedsystem, Austria) served for analysis. Tissue homogenates were applied to a 96-well plate pre-coated with a rat IL-6 antibody. Biotinylated conjugates and streptavidin-HRP were added to bind the first antibody. Finally, tetramethyl-benzidine was applied to form a purple-colored complex, the absorbance read at OD of 450 nm.

### 2.17. Tumor necrosis factor (TNF)-α

Our study used a rat TNF-α ELISA kit (catalog no. ERT2010-1, ASSAYPRO, USA). Briefly, plasma sample was added to a 96-well plate pre-coated with a biotinylated antibody. TNF-α protein of a sample was detected at O.D. 450 nm after adding streptavidin-peroxidase conjugate and peroxidase enzyme substrate.

### 2.18. CYP 4A protein expression

A gram of liver tissue homogenized in 4 mL 0.01 M phosphate buffer was centrifuged (3000 rpm) for 15 min. Supernatant was aspirated and centrifuged (at 10,000 ×*g* for 30 min and 32,000 ×*g* for 60 min) to acquire liver microsomes. After adjusting protein content, sample underwent electrophoresis: proteins of separation gel transferred to polyvinylidene difluoride membranes incubated with CP 4A monoclonal antibody, second antibody added with color-presenting agent to assess a group’s protein amount. Extent of CYP 4A protein expression on membrane was rated by Image Gauge software (Ver.4.01, Science Lab 2001, Fujifilm, Japan).

**Table 2:** Growth, food intake, and liver weight of animals during the experiment.

	N	HFD	RN	1×AP	2×AP
Body weight (gw)					
Week 8	191.1± 2.7	189.7± 2.8	187.2± 4.6	184.1± 2.6	193.1± 1.4
Week 12	427.9± 12.0^b^	496.7± 16.0^a^	458.7± 20.0^ab^	460.3± 11.0^ab^	427.5± 11.2^b^
Food intake (gw/d)	37.6± 0.3^a^	35.3± 1.2^a^	35.1± 1.9^a^	36.9± 0.7^a^	35.7± 0.8^a^
Food efficiency (%)	14.3± 0.6^b^	19.1± .2^a^	17.7± 1.1^ab^	16.4± 0.2^ab^	14.8± 1.8^b^
Liver weight (gw)	11.4± 0.4^c^	22.0± 1.3^a^	14.6± 0.6^b^	17.0± 0.6^b^	14.9± 0.8^b^
Hepatosomatic index (%)	2.6± 0.1^d^	4.4± 0.2^a^	3.2± 0.1^c^	3.7± 0.1^b^	3.4± 0.1^bc^

Data presented as mean ± SEM.

Superscripts represent statistically significant differences among groups. *p* <0.05 N: normal dietary group, HFD: high fat diet group, RN: normal diet revert group, 1×AP, and 2×AP: normal diet recovery supplemented with 0.75%(w/w) and 1.5% (w/w) AP. Hepatosomal index: liver weight/body weight

Liver tissue was removed and perfused with 0.9% normal saline,

largest lobule sectioned and fixed in 10% formaldehyde,

### 2.19. Histopathological observations

Liver tissue was removed and perfused with 0.9% normal saline, largest lobule sectioned and fixed in 10% formaldehyde, paraffin-embedded sections stained with hematoxylin and eosin (H& E). Fat droplets and neutrophil infiltration observed were interpreted by criteria of Brunt and colleagues [17]. Fat accumulation thus had three levels: no fat droplets occupying liver section as grade 0, and with <33%, 33%~66%, and >66% of fat droplets occupying liver section as grades 1, 2, and 3 fatty liver, respectively. As for inflamed liver, Brunt and colleagues designated grade 1 inflammation as 1 or 2 foci of neutrophil infiltration per field, while 2~4 or 4+ foci/field in liver biopsy indicated grade 2 and 3 hepatitis, respectively.

### 2.20. Statistical analysis

Data were processed with SPSS18.0 software SPSS, Chicago, IL) and expressed as mean ± SEM. Within-group comparisons were performed by one-way analysis of variance (ANOVA) followed by Duncan’s multiple-range test. Pearson’s correlation test was used for correlation analysis; *p* value <0.05 indicated statistical significance.

## 3. Results

### 3.1. Feed efficiency and animal growth

Table 2 depicts growth and feed efficiency of animals fed a high-fat diet as significantly higher than those of any other group. Subsequently, only the 2×AP group had significantly recovered food efficiency induced by high-fat diet by the end of the study. Liver weight and hepatosomatic index (liver weight/body weight) of animals that reverted to normal fat diet (RN, 1×AP, and 2×AP groups) were all significantly lower than those of the HFD group. However, levels in these groups had not returned to normal by the end of the study.

### 3.2. Biochemical data

As seen in Table 3, AST, ALT, plasma triglyceride, total cholesterol (TC), VLDL-C, LDL-C, FFAs, and lipid peroxidation levels all rose after eight weeks of high-fat induction, whereas HDL-C level significantly dropped. After returning to a normal-fat diet for four weeks, only plasma TC and LDL-C levels had decreased, while the HDL-C level slightly increased. APE supplementation with a normal diet produced obvious improvements in all blood lipid levels and marker of lipid peroxidation. For blood sugar control, APE supplement improved more than the dietary recovery group in fasting blood sugar and HOMA-IR (Table 4). Amounts of inflammatory markers, TNF-α and IL-6, in plasma were also rated. Data showed prominent changes on both TNF-α and IL-6 plasma levels after APE supplementation (*p* <0.05). For hepatic lipids, reverting to normal diet was definitely an effective way to regress. Still, AP supplementation further decreased cholesterol level below that of the RN group; 2×AP group displayed even lower hepatic triglycerides, which had almost reverted to normal.

**Table 3:** Results of AST, ALT , plasma lipids and lipid peroxidation before and after the AP supplementation

	N	HFD	RN	1×AP	2×AP
AST (U/L)					
Week 8	68.0 ± 3.6^b^	133.8 ± 16.6^a^	126.8 ± 23.8^a^	133.8 ± 8.0^a^	124.5 ± 19.1^a^
Week 12	71.1 ± 1.2^b^	113.0 ± 19.1^a^	87.3 ± 8.0^ab^	80.1 ± 6.2^b^	79.2 ± 4.4^b^
ALT (U/L)					
Week 8	37.3 ± 0.9^b^	57.7 ± 4.2^a^	52.7 ± 2.8^a^	65.5 ± 7.8^a^	62.2 ± 3.8^a^
Week 12	31.4 ± 1.4^b^	92.9 ± 26.0^a^	45.8 ± 3.3^ab^	47.3 ± 5.2^ab^	41.6 ± 5.1^b^
Triglyceride (mg/dL)					
Week 8	37.3 ± 1.2^b^	57.8 ± 5.3^a^	51.5 ± 3.0^a^	50.8 ± 2.9^a^	54.2 ± 3.8^a^
Week 12	21.0 ± 2.3^b^	40.9 ± 2.8^a^	38.1 ± 2.8^a^	27.6 ± 4.3^b^	23.1 ± 1.4^b^
TC (mg/dL)					
Week 8	46.7 ± 2.0^b^	69.1 ± 6.9^a^	64.2 ± 2.8^a^	69.8 ± 5.0^a^	69.0 ± 2.9^a^
Week 12	46.3 ± 3.9^c^	76.7 ± 2.9^a^	58.4 ± 3.3^b^	49.4 ± 2.4^bc^	48.4 ± 3.9^bc^
VLDL-C (mg/dL)					
Week 8	26.7 ± 1.5^b^	47.8± 5.6^a^	43.0 ± 3.0^a^	46.2 ± 6.0^a^	45.7 ± 4.4^b^
Week 12	16.8 ± 2.5^c^	29.3 ± 2.7^a^	24.6 ± 1.6^ab^	22.8 ± 1.7^bc^	20.1 ± 1.6^bc^
LDL-C (mg/dL)					
Week 8	5.3 ± 0.6^b^	23.2 ± 4.8^a^	22.3 ± 3.2^a^	18.5 ± 3.3^a^	24.5 ± 4.4^a^
Week 12	16.1 ± 0.6^c^	38.0 ± 3.2^a^	23.6 ± 2.5_b_	18.4 ± 1.4^bc^	18.4 ± 1.4^bc^
HDL-C (mg/dL)					
Week 8	14.2 ± 0.7^a^	6.0 ± 1.1^b^	7.0 ± 1.0^b^	7.8 ± 0.8^b^	7.2 ± 1.4^b^
Week 12	13.0 ± 1.0^a^	5.2 ± 0.8^c^	8.3 ± 0.8^b^	13.2 ± 1.3^a^	13.2 ± 1.3^a^
FFA (nmol/μL)					
Week 8	0.36 ± 0.02^b^	0.66 ± 0.11^a^	0.58 ± 0.05^a^	0.60 ± 0.23^a^	0.59 ± 0.08^a^
Week 12	0.32 ± 0.07^b^	0.56 ± 0.18^a^	0.48 ± 0.12^ab^	0.36 ± 0.13^b^	0.32 ± 0.06^b^
MDA (μM)					
Week 8	14.9 ± 1.5^b^	21.5 ± 0.6^a^	20.6 ± 0.9^a^	21.1 ± 0.9^a^	20.8 ± 0.9^a^
Week 12	17.0 ± 0.9^b^	22.6 ± 1.4^a^	19.7 ± 0.5^ab^	19.5 ± 1.6^ab^	16.8 ± 2.7^b^

Data presented as mean ± SEM.Superscripts represent statistically significant differences among groups. *p* <0.05 N: normal dietary group, HFD: high fat diet group, RN: normal diet revert group, 1×AP, and 2×AP: normal diet recovery supplemented with 0.75%(w/w) and 1.5% (w/w) AP.

### 3.3. Hepatic lipids, antioxidative status, and CYP 4A protein expression

**Fig. 1:**
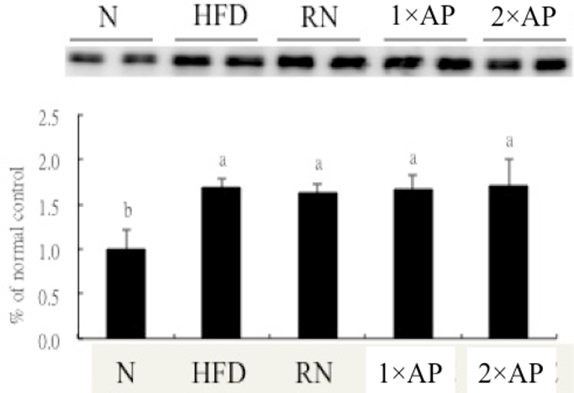
CYP4A protein expression in each group. (n=8) Different symbols on each bar represented significantly different within groups, *p* <0.05. N, normal dietary group; HFD, high-fat diet group; RN, normal diet reversion group; 1× and 2× AP, normal diet recovery supplemented with 0.75% (w/w) and 1.5% (w/w) of the water extract of *Auricularia polytricha* , respectively.

A high-fat diet dramatically decreased SOD, GPx, and GR activities with higher cholesterol, triglyceride and lipid peroxidation (MDA) levels. Upon reversion to normal diet, all enzyme activities improved. With further addition of 1.5% AP, GPx and GR activities even returned to normal, which saved vitamin E consumption. Serum vitamin C and E concentrations were kept under AP supplementation. Even hepatic vitamin E level might not be fully recovered by normal diet plus AP intervention, vitamin E levels in 1×AP and 2×AP groups were significantly higher than HFD group. CYP 4A is another identical biomarker for NAFLD. As seen in Figure 1, CYP 4A protein was highly expressed in all experimental groups fed the high-fat diet. This induction continued to the end of the experiment.

**Table 4:** End-point plasma glucose control, inflammatory markers, hepatic lipids, and oxidative enzyme activities.

	N	HFD	RN	1 × AP	2 × AP
FBS (mg/dL)	139.0 ± 7.1^b^	171.3 ± 5.6^a^	161.6 ± 8.2^a^	141.0 ± 2.1^b^	133.6 ± 7.4^b^
Insulin (μg/L)	0.21 ± 0.04^b^	1.20 ± 0.25^a^	0.38 ± 0.20^b^	0.29 ± 0.07^b^	0.22 ± 0.04^b^
HOMA-IR	1.7 ± 0.4^b^	7.9 ± 2.4^a^	4.2 ± 2.5^ab^	2.5 ± 0.6^b^	1.9 ± 0.4^b^
Vit E (μg/mL)	20.8 ± 1.0^a^	15.8 ± 0.7^b^	17.7 ± 1.2^ab^	20.0 ± 1.7^a^	21.2 ± 1.9^a^
Vit C (μM)	453.9 ±8.0^bc^	388.3 ± 12.7^d^	414.0 ± 17.0^cd^	476.4 ± 27.4^ab^	532.9 ± 37.5^a^
TNF-α (ng/mL)	1.8 ± 0.0^bc^	3.2 ± 0.4^a^	2.7 ± 0.5^ab^	1.2 ± 0.2^c^	1.2 ± 0.2^c^
IL-6 (ng/mL)	21.2 ± 2.2^b^	48.8 ± 5.8^a^	34.8 ± 7.2^ab^	25.1 ± 3.2^b^	23.0 ± 2.8^b^
Hepatic lipids					
cholesterol (mg/g)	2.7 ± 0.2^c^	28.7 ± 3.5^a^	12.0 ± 2.6^b^	4.9 ± 1.1^c^	5.5 ± 1.8^c^
triglyceride (mg/g)	4.5 ± 0.2^c^	19.1 ± 1.4^a^	8.8 ± 0.6^b^	8.4 ± 0.9^b^	6.4 ± 0.8^bc^
Hepatic oxidative status					
SOD (U/mg)	210.0 ± 12.0^a^	142.2 ± 11.9^b^	187.3 ±11.0^ab^	172.9 ± 32.8^ab^	206.1 ± 34.2^a^
GR (nmol/min/mg)	20.2 ± 3.3^a^	10.9 ± 1.2^b^	12.5 ± 2.1^ab^	14.9 ± 2.4^ab^	19.8 ± 3.5^a^
Catalase (nmol/min/mg)	2032.6 ±384.1^b^	3111.8 ± 250.2^a^	1664.0 ± 173.2^b^	1545.6 ± 166.4^b^	1804.4 ±162.4^b^
GPX (nmol/min/mg)	605.8 ± 85.5^a^	351.9 ± 16.0^c^	344.9 ± 23.4^c^	398.7 ± 14.7^bc^	511.8 ± 46.7^ab^
Vit E (μg/g)	63.4 ± 7.8^a^	14.1 ± 2.0^d^	18.8 ± 2.2^cd^	29.8 ± 3.4^bc^	34.2 ± 3.1^b^
MDA (μmol/g)	687.3 ± 62.2^c^	1433.5 ± 59.8^a^	933.7 ± 43.6^b^	740.1 ± 45.9^c^	699.6 ± 58.1^c^

Data presented as mean ± SEM.

Superscripts represent statistically significant differences among groups. *p* <0.05 N, normal dietary group; HFD, high-fat diet group; RN, normal diet reversion group; 1× and 2× AP, normal diet recovery supplemented with 0.75% (w/w) and 1.5% (w/w) of *Auricularia polytricha* water extract, respectively.FBS, fasting blood sugar; HOMA-IR, homeostatic model assessment- insulin resistance; TNF, tumor necrosis factor; IL, interleukin; SOD, superoxide dismutase; GR, glutathione reductase; GPx, glutathione peroxidase; MDA, malondialdehyde.

### 3.4. Histological observation

**Fig. 2:**
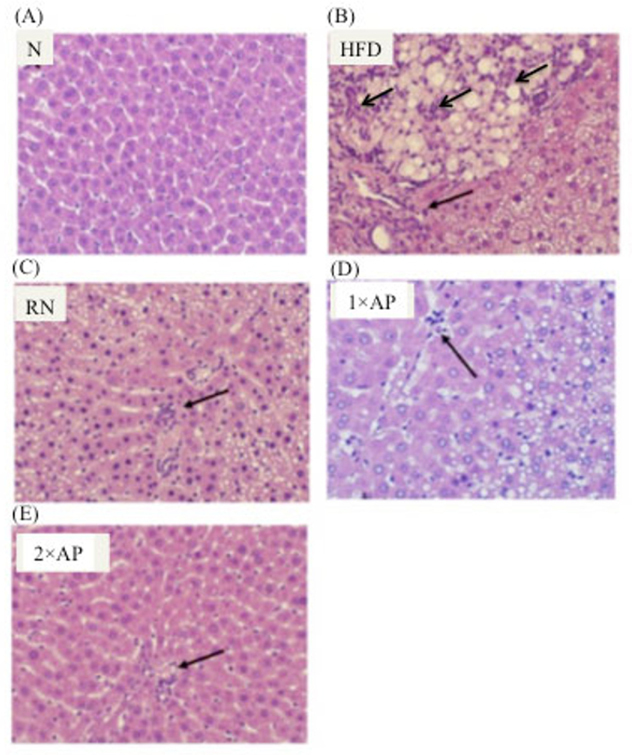
H & E stain of hepatic tissue sections in the groups. Pictures were taken under 400 times magnification. Interpreting by Brunt’s method, (A) grade 0 fatty liver and inflammation (B) grade 3 fatty liver and grade 2-3 inflammation (C) grade 2 fatty liver and grade 1-2 inflammation (D) grade1-2 fatty liver and grade 0-1 inflammation (E) grade 0-1 fatty liver and grade 0-1 inflammation. The arrow-pointing area indicated neutrophil infiltration foci in the tissue section.

Histological data also proved efficacy of AP at hepatoprotection. According to Brunt and colleague’s assessment method, the HFD group exhibited grade 3 fatty liver and grade 2~3 inflammation, while the normal control group belonged to grade 0 (Fig. 2). After reverting to normal diet, hepatic tissues of animals were grade 2 fatty livers and grade 1~2 inflammation. Improvement was even better with AP supplementation. Figure 1 shows animals in 1×AP group with grade 1~2 fatty liver and grade 0~1 inflammation versus 2×AP group with both fatty liver and inflammation grade 0~1. Masson’s trichrome stain indicated fibrotic signs in hepatic tissues, but there were no fibrotic indications in livers from animals subsequently fed high-fat diet for 12 weeks (data not shown).

## 4. Discussion

Many factors contribute to fatty liver: methionine-choline deficiency, alcohol, insulin resistance, hepatotoxins, lack of leptin or its receptor. Various animal models of NAFLD were devised based on those factors, but few matched most of the pathological symptoms of NAFLD cases; some showed inconsistent clinical evidence. Methionine-choline-deficient diet caused severe body weight loss not commonly seen in NAFLD patients; the same diet with additional injection of sodium nitrate directly resulted in skipping the process of hepatic necrosis. Liquid diet containing 71% fat did not fit dietary fatty acid pattern of NAFLD patients. After hypercaloric diet with 37% corn oil, animals did not have the same protein expressions of CYP2E1 and Peroxisome proliferator activated receptors (PPAR)-α. Overall, an animal model fed a combination of 88% chow diet (w/w), 10% lard (w/w), and 2% cholesterol (w/w) had strong phenomenological similarities to NAFLD clinical cases. Our study’s model successfully induced visceral obesity, high plasma FFA levels, hyperlipidemia, and liver CYP4A (data not shown) and CYP2E1 expression. Dramatic changes in AST and ALT are known signatures of liver disease. One study showed that ALT changed more than AST in NAFLD patients; fibrosis was initiated when greater AST changes occurred [18]. In this study, 12-week high-fat diet induced greater changes in ALT than AST activity. The model had consistent changes with clinical findings of NAFLD.

Two hits of lipid deposition and free radical formation describe pathological process of NAFLD, for which symptoms are reversible with proper nutritional adjustment. With high-fat intervention, insulin resistance (HOMA-IR) was induced, stimulating FFA release and transport to the liver that caused triglyceride storage therein. The high-fat diet also caused dysregulation of cholesterol transport. Although changes in plasma AST and ALT levels were not more obvious than those in the normal group, addition of AP made much greater progress in elevating insulin sensitivity and lipid metabolism than those with normal dietintervention, insulin resistance (HOMA-IR) was induced, stimulating FFA release and transport to the liver that caused triglyceride storage therein. The high-fat diet also caused dysregulation of cholesterol transport. Although changes in plasma AST and ALT levels were not more obvious than those in the normal group, addition of AP made much greater progress in elevating insulin sensitivity and lipid metabolism than those with normal diet.

When we discuss health-promoting effect of AP, polysaccharides are always addressed as chief active compounds. According to our laboratory data, the AP had 90% dietary fiber, including 74.0% soluble and 22.6% insoluble fibers; thus its action cannot be neglected. According to prior studies, about 5~10% (w/w) fiber in the diet, an average of 1~3 g fiber intake, can effectively improve symptoms of fatty liver disease by insulin regulation, antioxidation, and lipid-lowering action [19-21]. Effect of high fibers could also be shown as decreased food efficiency in our study. While it successfully regulates and decreases all the above-cited categories, 10% β-glucan did not decrease TNF-α expression in obese Zucker rats [22]. Compared to the normal (RN) group, extra fiber content in AP groups were no more than 0.4725 g (with average intake of 0.525 g of the AP), and lipid-lowering, glucose-homeostatic, antioxidative and also anti-inflammatory effects were seen in the study. These phenomena imply that the power of AP might emanate from fiber content and depend on its abundant polyphenols, especially tannins. Main phenols in AP were gallic acid, tannic acid, and protocatechuic acid [13]. Tannins in AP, rarely discussed in previous studies, are first emphasized our study. However, anti-inflammatory-related effects of phenols were often investigated. Other than raising GSH levels and lowering IL-6 and TNF-α, above-mentioned AP phenols might work via superoxide clearance and monocyte chemotactic protein (MCP)-1 suppression to implement antioxidative and anti-inflammatory action [23-25]. Functional components other than dietary fiber in AP were described in our study; laboratory analysis showed content of each hypothetically active component in AP not predominant among known natural herbs. Perhaps extraction produced specific combination that showed efficacy of improving NASH pathology, or actual active component remains unknown and needs further studies for elucidation.

Most dietary supplements or functional foods have good lipid-lowering effect, albeit potency too strong to affect HDL-C level. Balanced diet in the study reversed lipoprotein distribution but did not cause it to return to a normal level. A previous study discussed lipid-regulating effect of AA; results showed it lowering TC and LDL-C without affecting HDL-C level [26, 27]. Yet AP supplementation exerted its influence on lipoprotein metabolism and strongly elevated HDL-C. Higher amount of phenols in AP than in AA may elevate HDL-C by activating lipoprotein lipase activity [28]. Active components in AP may possess a CETP-inhibiting action to increase HDL-C [27], further research must clarify. Our results indicate AP as an ideal health food material for lipid regulation.

As for antioxidative status, high-fat diet for eight weeks yielded high oxidative status, as indicated by decrease in low-oxidative marker enzyme, GPx, and high induction of high-oxidative marker enzyme, catalase. Strictly normal diet failed to improve oxidative status or inflammatory biomarkers. Mushrooms have abundant antioxidants (phenolic compounds, polysaccharides, nicotinic acid, ergosterols, triterpenes); activities of most antioxidative enzymes like GR, GPx, and SOD in the 2×AP group had recovered. High phenol compounds and tannins in AP reduced consumption of tocopherols and ascorbic acid, which remained as antioxidative nutrients in a hyperoxidative body. CYP4A joined ω-oxidation in microsomes. Expression of CYP4A increases when peroxisomal lipid peroxidation occurs in mitochondria [29, 30]. Under PPAR-α stimulation, CYP4A regulates long-chain fatty acid oxidation, and elevates hydrogen peroxide to cause cell damage and fatty liver formation. Overexpression of PPAR-α was observed in NASH patients [30]. In the present study, benefit of CYP 4A was not seen in normal-diet or AP-supplemented groups. Further studies must clarify action of AP on microsomal fatty acid oxidation.

Clinical studies show NASH patients with phenomenally high TNF-α expression than those with other fatty liver disease [31]. TNF-α released by visceral fat accelerates NAFLD progression and activates its receptor to cause hepatic fat to accumulate. Increased oxidized FFAs induce a hepatic kinase (IKKβ) pathway, then secrete TNF-α and IL-6. Eventually, both cytokines might postpone the signaling pathway, triggering insulin resistance [32]. This portends AP effectively removing two hits in a NASH animal model. If we convert effective dosage in the study for human usage, 1.5 g/kg BW in rats might be equivalent to 0.24 g/kg BW for humans. Taking extraction rate into consideration, a 60 kg man would ingest 144 g dry AP powder, though crude powder without proper purification had lower polyphenol, tannin, and flavone levels. APE shows definite potential for new functional food ingredients.

## 5. Conclusions

This study proved supplementation with AP effectively mitigating two-hit factors of NAFLD and postponing disease progression. Future research can focus on health benefits toward metabolic syndrome, type 2 diabetes, and related metabolic disorders. Studies of active components in AP are also needed for health food development.

## Acknowledgments

Financial support of Council of Agriculture, Executive Yuan, Taiwan (101103) is thankfully acknowledged.
